# Identification of Leaf Rust Resistance Genes in Selected Egyptian Wheat Cultivars by Molecular Markers

**DOI:** 10.1155/2014/574285

**Published:** 2014-01-06

**Authors:** I. A. Imbaby, M. A. Mahmoud, M. E. M. Hassan, A. R. M. Abd-El-Aziz

**Affiliations:** ^1^Plant Pathology Research Institute, Agricultural Research Center, Giza 12619, Egypt; ^2^Botany and Microbiology Department, College of Science, King Saud University, Riyadh 1145, Saudi Arabia

## Abstract

Leaf rust, caused by *Puccinia triticina* Eriks., is a common and widespread disease of wheat (*Triticum aestivum* L.) in Egypt. Host resistance is the most economical, effective, and ecologically sustainable method of controlling the disease. Molecular markers help to determine leaf rust resistance genes (*Lr* genes). The objective of this study was to identify *Lr* genes in fifteen wheat cultivars from Egypt. Ten genes, *Lr13*, *Lr19*, *Lr24*, *Lr26*, *Lr34*, *Lr35 Lr36*, *Lr37*, *Lr39*, and *Lr46*, were detected in fifteen wheat cultivars using various molecular markers. The most frequently occurring genes in fifteen Egyptian wheat cultivars were *Lr13*, *Lr24*, *Lr34*, and *Lr36* identified in all the cultivars used, followed by *Lr26* and *Lr35* (93%), *Lr39* (66%), *Lr37* (53%), and *Lr46* (26.6%) of the cultivars, and finally *Lr19* was present in 33.3% of cultivars. It is concluded that there was a good variation in *Lr* genes carried by wheat cultivars commercially grown in Egypt. Therefore, strategies for deploying resistance genes to prolong effective disease resistance are suggested to control wheat leaf rust disease.

## 1. Introduction

Rusts are the most devastating fungal diseases posing a threat to wheat production worldwide. Leaf rust, caused *Puccinia triticina* Eriks., is a major disease in most of the wheat-growing areas [1]. In Egypt, leaf rust is the most common and important wheat disease. It caused severe losses in grain yield which reached 23% [2] and losses in epidemic years reached up to 50% [3]. The most environmentally sound, low cost method of controlling leaf rust is to breed and grow resistant wheat varieties. So far over 60 leaf rust resistance genes, that is, *Lr* genes, have been identified and localized on the wheat chromosomes [4]. Resistance genes are expressed at seedling stage (qualitative resistance genes) than at adult plant (quantitative resistance genes). Certain adult plant resistance genes like *Lr34* and *Lr46* are very important for breeding because they proved to confer durable resistance over a long period of time in different environments, as well as against diverse pathotypes of the fungus [5].

The effectiveness of resistance genes depends on the composition of the pathogen populations. As this changes dynamically, new pathotypes virulent to the given resistance genes multiply from time to time, so the resistance of a variety is not a constant trait. A variety carrying a single resistance gene mat becomes susceptible within a short time. The postulation of resistance genes is traditionally carried out using rust isolates with known virulence [6] but this procedure is extremely time, space, and labour intensive and cannot be employed if no different fungal isolates are available. In many cases resistance genes can only be identified using molecular markers [7]. Over the last 15 years many efficient markers for leaf rust resistance genes have been described. The molecular markers most closely linked to *Lr* genes are based on the PCR technique, as the majority of these can be applied relatively easily in wheat breeding programmers.

Molecular markers are used for two purposes in resistance breeding: (1) to monitor the incorporation of designated resistance genes or QTLs into elite wheat genotypes (i.e., MAS, marker-assisted selection) and (2) to identify resistance genes in varieties and lines where the genetic background is unknown (i.e., gene detection). A great deal of information on postulated leaf rust resistance genes has been collected from countries (including Australia, Canada, China, India, Pakistan, South Africa, and USA) where wheat is a major crop [8–12]. Little information is available on *Lr* genes present in Egyptian wheat cultivars [13, 14].

Objective of this study is to identify genes for resistance to leaf rust disease in selected Egyptian wheat cultivars.

## 2. Materials and Method

### 2.1. Plant Material

Fifteen wheat cultivars were used. These cultivars were tested for leaf rust disease under green house at seedling and adult plant stages. Fifteen Egyptian wheat cultivars and seven Near-Isogenic Thatcher lines (NILs) were tested for wheat leaf rust for their reaction to leaf rust. The wheat cultivars include Giza cultivars (163, 164, 165, 167, and 168), Sakha cultivars (8, 61, 69, 92, and 94), Sids cultivars (1 and 12) and Gemmeiza cultivars (7, 9, and 10). The selected Thatcher NILs were *Lr13*, *Lr19*, *Lr24*, *Lr26*, *Lr34*, *Lr37*, *Lr36*, and *Lr49*.

### 2.2. Disease Assessment

#### 2.2.1. Seedling Stage

The cultivars to be tested were planted in 7 cm square plastic pots. Four cvs were planted per pot with 10–15 seeds per cv planted in each corner of the pot. Plants were grown in rust-free greenhouse until inoculation. At 7 days after planting when first leaves were fully expanded, the seedlings were gently rubbed between moist fingers and then sprayed with tap water using atomizer in the inoculation chamber, then inoculated by spraying them with a suspension of urediospores in a light mineral oil carrier. Inoculum concentration was normalized to 2-3 mgmL^−1^ [15]. The oil was allowed to evaporate from the leaves for 30–60 min, and the seedlings were placed overnight in a dew chamber at 17°C. They were then transferred to a greenhouse with mean temperature approximately 20-21°C. At 14 days after inoculation, the cvs were scored for infection type (IT) according to the scale of [16], where 0: nearly immune; 1: very resistant; 2: moderately resistant; 3: moderately resistant to moderately susceptible; and 4: very susceptible.

#### 2.2.2. Adult Stage

The aforementioned cvs were sown in 30 cm square diameter pots. Each cv was planted in each pot, and four pots were planted for each cv as replicates. 75 days after planting (prebooting stage) [17], the plants were inoculated as mentioned before. After incubation, the plants were transferred onto the greenhouse benches. The disease severity (%) was recorded as the area of leaf covered with rust pustules according to the method adopted by [16]. Moreover, the particular cvs were planted in 25 cm square diameter pots and were left till tillering stage [17] then harvested for gene identification by using molecular markers.

### 2.3. DNA Extraction

DNA was isolated from 50 of the varieties (each) using Qiagen kit for DNA extraction. The extracted DNA was dissolved in 100 ul of elution buffer. The concentration and purity of the obtained DNA were determined by using “Gen Qunta” system, pharmacia Biotech. The purity of the DNA for all samples was between 90 and 97% and the ratio between 1.7 and 1.8 concentrations was adjusted at 6 ng/ul for all samples using TE buffer pH 8.0.

### 2.4. Detection of *Lr* Genes by Molecular Markers

Thirty ng from the extracted DNA, 0.25 *μ*M of each primer of its and 0.40 *μ*M from each specific primer (10 primers) were used for amplification reaction. The PCR mixture contained PCR beads tablet (manufactured by Amesshan Pharmacia Bio-tech) which contained all of the necessary reagents except the primer and the DNA to be used. The total volume was completed to 25 *μ*L using sterile distilled water. The sequences of the used primers and size fragment are present in Table 3. Amplifications were performed in T-gradient thermocycler (Biometra, Germany). Sequences of primers are listed in Table 1. Amplification parameters for all primer sets used are presented in Table 2.

### 2.5. Electrophoresis

Amplification products were separated with 2% agarose gel (Applichem, Germany) in 1x TBE buffer and stained with ethidium bromide (0.5 *μ*g/mL). The 10 *μ*L PCR products were combined with 3 *μ*L of loading buffer, which was added to prepare samples for agarose gel electrophoresis. PCR products were electrophoresed at 75 volt using an electrophoresis unit (WIDE mini-sub cell GT Bio-Rad), and determined with UV transilluminator.

### 2.6. Gel Analysis

The DNA was scanned for band Rf using gel documentation system (AAB Advanced American Biotechnology 1166E. Valencia Dr. Unit 6 c, Fullerton CA 92631). The different MW bands were determined against PCR marker Promega G 4521 50 bp DNA step ladder and Amresco 100 bp k180 by unweighted pair-group method based on arithmetic mean (UPGMA).

## 3. Results 

### 3.1. Tested Cultivars and Resistance to Leaf Rust Disease

Resistance of the fifteen wheat cultivars to leaf rust isolates at seedling and adult plant stages is shown in Table 3. Some cultivars, that is, Giza 168, Sakha 94, and Gemmeiza 9, showed resistance at both stages; meanwhile, Giza 163, Giza 164, Giza 165, Sakha 69, and Gemmeiza 10 were resistant at seedling but susceptible at adult plant stage. The rest of the cultivars, that is, Sakha 61, Sakha 92, Sids 1, and Gemmeiza 7, were susceptible at both stages.

### 3.2. Leaf Rust Resistance Gene Efficacy

The efficiency of wheat genotypes carrying designated *Lr* genes, that is, *Lr 13*, *Lr19*, *Lr24*, *Lr26*, *Lr34*, *Lr35*, *Lr36*, *Lr37*, *Lr39*, and *Lr46*, was estimated at seedling and adult plant stages (Table 4). The result indicated that none of the tested *Lr* genes were effective. At adult stage (under field conditions) *Lr34* (efficacy 100%) was the most effective gene, followed by *Lr39* (85%), then *Lr19* (75%) and *Lr46* (60%). *Lr*'s 13, 24, 26, and 37 were not effective under the Egyptian conditions.

### 3.3. Detection of *Lr* Genes with Molecular Markers

**Figure 1:**
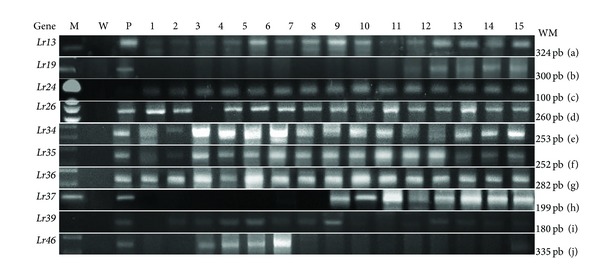
PCR amplification of 15 cultivars genomic DNA using ten *Lr* molecular marker. Lane M, 100pb marker; lane W, water as negative control; P, positive control; lane 1 Giza 163 cv, lane 2 Giza 164; lane 3, Giza 165; lane 4, Giza 167; lane 5, Giza 168; lane 6, Sakha 8; lane 7, Sakha 61, lane 8, Sakha 69; lane 9, Sakha 92; lane 10, Sakha 94; lane 11, Sids 1; lane 12, Sids 12; lane 13, Gemmeiza 7; lane 14, Gemmeiza 9, lane 15, Gemmeiza 10. (a) *Lr13*, (b) *Lr19*, (c) *Lr24*, (d) *Lr26*, (e) *Lr34*, (f) *Lr35*, (g)* Lr36*, (h) *Lr37*, (i) *Lr39*, and (j) *Lr46*.

15 wheat cultivars, Giza (163, 164, 165, and 168), Sakha (8, 61, 69, 92, and 94), Sedes (1 and 2) and Gemmeiza (7, 9, and 10), were examined by using molecular markers for ten *Lr* genes (*Lr13*, *Lr19*, *Lr24*, *Lr26*, *Lr34*, *Lr35*, *Lr36*, *Lr37*, *Lr39*, and *L 46*) against the fungal pathogen of wheat (Figure 1). The size of amplified marker fragment is shown in Table 5 as *Lr13*, 19, 24, 26, 34, 35, 36, 37, 39, and 46 for 324 bp, 300 bp, 100 bp, 260 bp, 253 bp, 252 bp, 282 bp, 199 bp, 180 bp, and 335 bp, respectively. Data presented in Table 5 illustrates leaf rust resistance genes identified in the used selected cultivars. using molecular markers. Genes *Lr13*, *Lr24*, *Lr34*, and *Lr36* were identified in all the cultivars used. *Lr26* was identified in 93% also, *Lr35* get same present, *Lr39* was identified in 66% of the materials followed by *Lr37* (53% of the materials). *Lr46 *was present in 26.6% of the cultivars and finally *Lr19* was present in 33.3% of cultivars.

## 4. Discussion

Survey for wheat leaf rust in Egypt during many growing seasons, 2000–2012, indicated the presence of the disease incited by *P. triticina* in different governorates. Most of diseased samples were collected from farmer fields and trap nurseries [13, 14, 18, 19]. One of the most important steps in breeding programs for rust resistance in wheat is the identification of the prevailing physiological races in the region. Such program will be successful if all physiological isolates of the disease are included [9].

In recent years developments in molecular marker techniques and marker identification have facilitated the spread of molecular-assisted selection (MAS). This is particularly true in the field of breeding wheat for leaf rust resistance, where PCR-based markers are already available for almost half of the 60 or more designated resistance genes and alleles. Furthermore, all the effective resistance genes designated so far can be traced in segregating progeny populations by means of MAS.

The genes *Lr13*, *Lr24*, *Lr34*, and *Lr36* were the most common resistance genes that could be identified in the cultivars. *Lr13* is probably the most widely distributed *Lr* gene in the world [20]. 58% of the European wheat genotypes tested carried *Lr13* alone or in combination [21]. The gene was once considered to confer durable adult plant resistance but is now ineffective in several countries including Mexico [8]. *Lr13* is still considered effective in combinations with other race-specific genes in Australia as the *Lr13*-virulent pathotype was avirulent on many other resistance genes [22]. However, in Egypt pathotypes contain virulence to *Lr13* in combination with virulence on several important resistance genes and many vars. that carries *Lr13* alone or in combination with other genes were susceptible in the field trails. As expected *Lr34* was found in all tested cultivars, although this gene alone is capable of reducing the level of infection to almost half, as reported by [23]; resistance that is both excellent and durable can only be achieved if *Lr34* is combined with 2 or 3 other genes [24].

*Lr24* and *Lr26* genes were identified in the tested cultivars but were not effective in Egypt. The resistance gene *Lr26* is present on the rye segment in a T1BL-1RS wheat-rye translocation. The cultivars “Brigadier,” “Florida,” “Haven” and “Toronto,” show infection types corresponding to *Lr26* and carry the T1BL-1RS translocation [25]. Moreover, it has become clear that virulence to *Lr26* exists in Northern Europe [26]. Gene *Lr37* showed intermediate resistance in Egypt. Gene *Lr37* confers mainly adult plant resistance and is difficult to detect in seedling tests. The cultivars that seemed to carry *Lr37* singly provided low seedling resistance and full adult plant resistance in Western Europe in 1996–1999 [22].

Most of the resistance genes included in the present study were detected in the Egyptian cultivars. The presence of *Lr13* and *Lr19* was confirmed by specific amplification of single fragments 324 and 300 bp. The resistance reaction to the rust pathotypes revealed the presence of *Lr19* gene. Similarly, rust resistance genes *Lr24* and *Lr26* resulted in the amplification of the expected fragments 100 and 260 bp. The other resistance genes *Lr34*, 35, 36, 37, 39 and 46 show specific amplification fragments of 253, 252, 282, 199, 180, and 335 bp, respectively. Leaf rust resistance gene *Lr19* has linkage with stem rust resistance gene Sr25 and a gene that causes yellowness of wheat flour [20].

The *Lr24* gene is known to be linked to the *Sr24* gene for resistance to stem rust, which is apparently effective against all races of stem rust [6] of study paving the way for marker-aided selection of rust resistance genes. The utility of such studies is further authenticated by other studies, where the presence of rust resistance genes was confirmed with molecular markers [27, 28]. Marker-assisted selection offers the opportunity to select desirable lines on the basis of genotype rather than phenotype, especially in the case of combining different genes in a single genotype. With the help of molecular marker, the pyramiding of leaf rust resistance genes, which are active at the seedling and/or adult stage, should facilitate more efficient breeding for durable resistance against this disease. The mechanism for durable resistance to leaf rust is poorly understood, but durability appears to be enhanced when genes are combined [29].

Experience gained so far suggests that markers flanking *Lr *genes can be used simply and effectively in marker-assisted backcross programmers. Nevertheless, as the linkage between markers and resistance genes is not complete, regular phenotypic monitoring will be required if satisfactory parental genotypes are to be selected. According to our earlier results [30] the ratio of false positive plants for the genes *Lr9*, *Lr24*, *Lr25*, and *Lr29* were 1.3, 4.0, 9.5, and 7.6%, respectively. However, molecular markers can prove the presence of the requested resistance gene in the genetic background and in the case of plants carrying adult plant resistance genes like *Lr35* and *Lr37* this is the only way to choose appropriate parents for crossing programmer. The use of MAS, whereby breeders select molecular markers linked to *Lr* genes, enables the pyramiding of more than one effective resistance gene. With the help of molecular markers, resistance genes are easy to detect in wheat varieties of unknown parentage. This information can then be used to design crossing programmers.

## Figures and Tables

**Table 1:** Sequences of the nucleotide primers used in this study.

*Lr* gene	Primer code	Sequence of primers (5′-3′)	Size of amplified marker fragment (bp)
13	13F	GTGCCTGTGCCATCGTC	324
	13R	CGAAAGTAACAGCGCAGTGA	[31]

19	19F	CATCCTTGGGGACCTC	300
	19R	CCAGCTCGCATACATCCA	[32]

24	24F	TCTAGTCTGTACATGGGGGC	100
	24R	TGGCACATGAACTCCATACG	[33]

26	26F	CATCCTTGGGGACCTC	260
	26R	CCAGCTCGCATACATCCA	[34]

34	34F	GTGAAGCAGACCCAGAACAC	253
	34R	GACGGCTGCGACGTAGAG	[35]

35	35F	AGAGAGAGTAGAAGAGCTGC	252
	35R	AGAGAGAGAGCATCCACC	[36]

36	36F	GCTGCATGAGCTCTGCAAT	282
	36R	TCTGTGAGGCATGACAGAA	[37]

37	37F	AGGGGCTACTGACCAAGGCT	199
	37R	TGCAGCTACAGCAGTATGTACACAAAA	[38]

39	39F	CCTGCTCTGCCCTAGATACG	180
	39R	ATGTGAATGTGATGCATGCA	[39]

46	46F	AGG GAAAAGACATCTTTTTTT TC	335
	46R	CGACCGACTTCGGGTTC	[35]

**Table 2:** Amplification parameters for all primer sets used.

*Lr* gene	Cycle condition
13	94°C 5 min., 30 cycles (94°C 1.5 min., 55°C 2 min., 72°C 1.5 min.), 72°C 5 min.
19	94°C 4 min., 40 cycles (92°C 1 min., 60° 1 min., 72°C 2 min.), 72°C 5 min.
24	94°C 5 min., 30 cycles (94°C 1.5 min., 55°C 2 min., 72°C 1.5 min.), 72°C 5 min.
26	94°C 2 min., 35 cycles (94°C 30 s., 63°C 2 min., 72°C 1.5 min.), 72°C 5 min.
34	94°C 5 min., 35 cycles (94°C 30 s., 65°C 2 min., 72°C 2 min.), 72°C 5 min.
35	94°C 10 min., 35 cycles (94°C 1 min., 54°C 1 min., 72°C 2 min.), 72°C 5 min.
36	94°C 5 min., 35 cycles (94°C 1 min., 57°C 1 min., 72°C 2 min.), 72°C 5 min.
37	94°C 10 min., 40 cycles (94°C 1 min., 55°C 1 min., 72°C 1 min.), 72°C 10 min.
39	94°C 4 min., 10 cycles (94°C 1 min., 64°C 1 min., 72°C 1 min.), 30 cycles 94°C 1 min., 55°C 1 min., 72°C 1 min.), 72°C 5 min.
46	94°C 4 min., 40 cycles (94°C 1 min., 58°C 1 min., 72°C 1 min.), 72°C 10 min.

**Table 3:** Wheat cultivars tested at seedling and adult plant stages for resistance to leaf rust disease.

Cultivar	Pedigree	Resistance to leaf rust disease	
		Seedling^a^	Adult^b^
Giza 163	T.aestivum/Bom/Ciano/3/Siete Cerros	1, 2	50S
Giza 164	Kavkas/Buho“s”//Kal/Bluebird=Verry#5	1	80S
Giza 165	Ciano/Maris Fundin//Mantaro	2, 3	90S
Giza 167	Au/Up 301//GII/Sx/3/Pew “s”/4/Mai “s”/Maya “s”//Pew	3	50S
Giza 168	MRL/BUC//Seri.CM93046-8M-0Y-0M-2Y-0B	2	20MR
Sakha 8	Indus/Norteno “s”	0, 1	10MSS
Sakha 61	Inia-RL 4220//Siete Cerros/Yaqui 50	3	50S
Sakha 69	Inia-RL 4220//Siete Cerros/Yaqui 50	2	5S
Sakha 92	Napo 63/Inia 66//Wren “s”	3	20S
Sakha 94	Opata/Rayon//KauzCMBW9043180-OTOPM-3Y-010M-010M-010Y-10M-015Y-0Y	1, 2	10MRMS
Sids 1	HD2172/Pavon “s”//1158. 57/Maya 74 “s”	3	80S
Sids 12	BUC//7C/ALD/5MAYA74/ON//1160-147/3/BB/GLL/4/CHAT:S′′/6/MAYA/VUL//CMH74A/4∗SX.SD7096-4SD-1SD-1SD-0SD.	—	—
Gemmeiza 7	CMH74A.630/5X82/3AgentCGM.4611-2GM-3GM-1GM0GM	4	20S
Gemmeiza 9	Ald“S”/Haus//CMH74A.630/SxCGM4583-5GM-1GM-0GM.	0, 1	10MRMS
Gemmeiza 10	Maya74“S”/ON/1160-147/3/Bb/G11/4/chat“S”/5/crow“S”CGM5820-3GM-1GM-2GM-0GM	1	10MSS

^a^0: nearly immune; 1: very resistant; 2: moderately resistant; 3: moderately resistant to moderately susceptible; and 4: very susceptible; ^b^rust severity (%); MR: moderately resistant; MS: moderately susceptible; S: susceptible.

**Table 4:** Efficacy % of the resistance genes for leaf rust disease at seedling and adult plant stages under the Egyptian conditions.

*Lr* gene	Efficiency %	
	Seedling	Adult
13	27.52	0.00
19	79.55	75.0
24	52.07	25.0
26	46.00	0.00
34	64.22	100.0
37	32.82	50.0
39	—	85.0
46	43.63	60.0

**Table 5:** Presence of resistance genes to leaf rust in the wheat cultivars used.

Cultivar	*Lr* genes									
	13	19	24	26	34	35	36	37	39	46
Giza 163	+	−	+	+	+	+	+	−	−	−
Giza 164	+	−	+	+	+	+	+	−	+	−
Giza 165	+	−	+	−	+	+	+	−	+	+
Giza 167	+	−	+	+	+	+	+	−	+	+
Giza 168	+	−	+	+	+	+	+	−	+	+
Sakha 8	+	−	+	+	+	+	+	−	−	+
Sakha 61	+	−	+	+	+	+	+	−	+	−
Sakha 69	+	−	+	+	+	+	+	+	+	−
Sakha 92	+	−	+	+	+	+	+	+	−	−
Sakha 94	+	−	+	+	+	−	+	+	−	−
Sids 1	+	+	+	+	+	+	+	+	−	−
Sids 12	+	+	+	+	+	+	+	+	+	−
Gemmeiza 7	+	+	+	+	+	+	+	+	+	−
Gemmeiza 9	+	+	+	+	+	+	+	+	+	−
Gemmeiza 10	+	+	+	+	+	+	+	+	+	−

(+) presence of gene; (−) absence of gene.
